# GSK621 Targets Glioma Cells via Activating AMP-Activated Protein Kinase Signalings

**DOI:** 10.1371/journal.pone.0161017

**Published:** 2016-08-17

**Authors:** Hong Jiang, Wei Liu, Shi-Kun Zhan, Yi-Xin Pan, Liu-Guan Bian, Bomin Sun, Qing-Fang Sun, Si-Jian Pan

**Affiliations:** 1 Department of Neurosurgery, Rui-Jin Hospital, Shanghai Jiao-Tong University School of Medicine, Shanghai, 200025, P.R. China; 2 Department of Stereotactic and Functional Neurosurgery, Rui-Jin Hospital, Shanghai Jiao-Tong University School of Medicine, Shanghai, 200025, P.R. China; Suzhou University, CHINA

## Abstract

Here, we studied the anti-glioma cell activity by a novel AMP-activated protein kinase (AMPK) activator GSK621. We showed that GSK621 was cytotoxic to human glioma cells (U87MG and U251MG lines), possibly via provoking caspase-dependent apoptotic cell death. Its cytotoxicity was alleviated by caspase inhibitors. GSK621 activated AMPK to inhibit mammalian target of rapamycin (mTOR) and downregulate Tetraspanin 8 (Tspan8) in glioma cells. AMPK inhibition, through shRNA knockdown of AMPKα or introduction of a dominant negative (T172A) AMPKα, almost reversed GSK621-induced AMPK activation, mTOR inhibition and Tspan8 degradation. Consequently, GSK621’s cytotoxicity in glioma cells was also significantly attenuated by AMPKα knockdown or mutation. Further studies showed that GSK621, at a relatively low concentration, significantly potentiated temozolomide (TMZ)’s sensitivity and lethality against glioma cells. We summarized that GSK621 inhibits human glioma cells possibly via activating AMPK signaling. This novel AMPK activator could be a novel and promising anti-glioma cell agent.

## 1. Introduction

Glioma is a common primary brain tumor, which among the most aggressive human malignancies [1]. Even with the development of modern treatments, the prognosis of metastatic and/or recurrent glioma is still extremely poor, and the overall survival is dismissal [1]. Late diagnosis, absence of specific markers, resistance of traditional therapy (radiation and temozolomide), the high potential of invasion and migration are all possible causes of its poor prognosis [2,3]. Therefore, our group [4,5,6,7] and others [8,9] are working on indentifying novel and important oncotargets of glioma, and exploring possible intervention strategies.

AMP-activated protein kinase (AMPK) plays a pivotal role in energy balance [10]. Yet, recent studies have proposed that this serine/threonine protein kinase could also be an important oncotarget [7,11]. Studies had shown that many anti-cancer drugs, including vincristine [12,13], taxol [14,15], temozolomide [16] and doxorubicin [17,18], can activate AMPK-dependent apoptosis to inhibit cancer cell growth. Our recent studies showed that gambogic acid induced glioma cell death via activating AMPK signalings [7]. Therefore, targeted-activation of AMPK could be a valuable strategy to inhibit glioma cells.

Thus far, many AMPK activators have been characterized, the majority of them activate AMPK via increasing the AMP: ATP ratio [19,20]. Yet, others increase AMPK activity by stimulating the phosphorylation of Thr-172 or by directly binding to AMPK subunits [19,20]. Recent research effects have developed a novel AMPK activator, named GSK621 [21]. In the present study, we tested the potential anti-cancer activity of GSK621 in glioma cells, the underlying the signaling mechanisms were also analyzed.

## 2. Materials and Methods

### 2.1. Chemicals and Reagents

Temozolomide (TMZ), AICAR and caspase inhibitors (z-DEVD-cho and z-VAD-cho) were purchased from Sigma-Aldrich Chemicals (St. Louis, MO). GSK621 was purchased from Selleck (Shanghai, China). All the antibodies utilized in this study were purchased from Cell Signaling Tech (Shanghai, China).

### 2.2. Cell Culture

Human glioma cell lines, U251MG and U87MG, as well as the HCN-1a human neuronal cell line were purchased from the Chinese Academy of Sciences Cell Bank. Glioma cells and HCN-1a cells were cultured as described [4,6,7]. Human primary astrocyte cultures were purchased from the iBS Cell Bank of Fudan University (Shanghai, China) [22]. The astrocytes were derived from the cerebral cortices of a single trauma patient. All the astrocytes were positive of glial fibrillary acidic protein (GFAP). Primary human astrocytes were maintained in astrocyte media as described [22].

### 2.3. Cell Viability Assay

As reported [4,6], the cell viability was tested by the MTT assay. Following the treatment of cells, 0.5 mg/mL MTT was added for 4 hours at 37°C. Afterwards, purple formazan salt crystals were dissolved by adding the solubilization solution (10% SDS, 0.01 M HCl). The absorption at 490 nm was measured on a multi-well plate reader [4,6].

### 2.4. Cell Death Detection

Following applied treatments, cells were harvested with trypsin/EDTA, suspended in PBS, and mixed with 0.4% trypan blue dye (Sigma). Viable cells maintained membrane integrity and did not take up trypan blue. Cells with compromised cell membranes took up trypan blue, and were counted as dead [6].

### 2.5. Clonogenicity Assay

As described in our previous studies [6,7], following applied GSK621 treatment, U87MG cells (5 × 10^3^ per dish) were resuspended in complete medium with 1% agar (Sigma, St. Louis, MO), which were then added on top of a pre-solidified 1% agar in a 100 mm culture dish. The medium was replaced every two days. After 8 days of incubation, the remain viable colonies were stained and manually counted.

### 2.6. Caspase-3 Activity Assay

Ac-DEVD-AMC (Peptides International, Louisville, KY) was utilized as the fluorogenic substrate for caspase-3. After applied treatments, cell lysates were incubated with Ac-DEVD-AMC at 37°C for 15 min. The activity of caspase-3 was measured following the cleavage of fluorogenic substrate excited at 370 nm by measuring the emission at 455 nm.

### 2.7. Quantification of Apoptosis by Enzyme-Linked Immunosorbent Assay (ELISA)

As described in our previous studies [6,7], following applied treatment, cell apoptosis was quantified by the Cell Apoptosis Histone DNA ELISA Detection Kit (Roche, Palo Alto, CA), according to the manufacturer's protocol [23].

### 2.8. Apoptosis FACS Analysis

After indicated treatment, cells were washed and fixed as described [6] and were resuspended in PBS containing RNase A (100μg/mL), propidium iodide (50μg/mL), Annexin V-FITC (50μg/mL) and 0.1% Triton X-100 [6]. Fluorescence-activated cell sorting (FACS) was performed to quantify the apoptotic population by Annexin V staining.

### 2.9. Western Blot Analysis

Cells were lysed in the described lysis buffer [4] on ice for 30 min, and the supernatants were collected. Equal amount of lysate samples (30 μg) was separated by SDS-PAGE gels, and were transferred to PVDF membranes. Following incubation of indicated primary antibodies and secondary antibodies, specific bands were visualized using enhanced chemiluminescence (ECL) reagents (Amersham Pharmacia Biotech, Piscataway, NJ) based on the molecular weight (MW). Band intensity was always quantified through total gray using the ImageJ software (NIH).

### 2.10. AMPKα shRNA Knockdown and Stable Cell Selection

The two lentiviral AMPKα short hairpin RNAs (shRNAs) were purchased from Santa Cruz Biotech (“AMPKα shRNA no.1”, Shanghai, China) and Genepharm (“AMPKα shRNA no.2”, Shanghai, China). Each shRNA was added to U87MG cells separately for 24 hours. Cell culture medium was then replaced by fresh medium for additional 24 hours. Stable clones expressing AMPKα shRNA were selected by puromycin (0.5 μg/mL, Sigma) for a total of 10 days. Control cells were infected with lentiviral scramble shRNA (Santa Cruz) [7]. AMPKα protein expression was always tested by Western blot in the resistant colonies.

### 2.11. AMPK Dominant Negative Mutation

The dominant negative AMPKα (DN-AMPKα, T172A) construct was designed and verified by Dr. Lu’s group at Nanjing Medical University [24]. For transfection, cells were seeded onto six-well plates with 60–70% of confluence in medium without antibiotics. DN-AMPKα cDNA (0.10 μg/mL) was transfected to the glioma cells through the Lipofectamine 2000 protocol [24], and stable cells were selected via neomycin (1.0 μg/mL, Sigma). Transfection efficiency was always verified via Western blot testing AMPK expression and activation.

### 2.12. Statistical Analysis

All statistics were calculated using SPSS 18.0 statistical software (SPSS, Chicago, IL). Descriptive statistics including mean and standard deviation (SD) along with one-way ANOVAs were applied to determine significant differences. p < 0.05 was considered significant.

## 3. Results

### 3.1. GSK621 Inhibits Human Glioma Cell Survival

**Fig 1:**
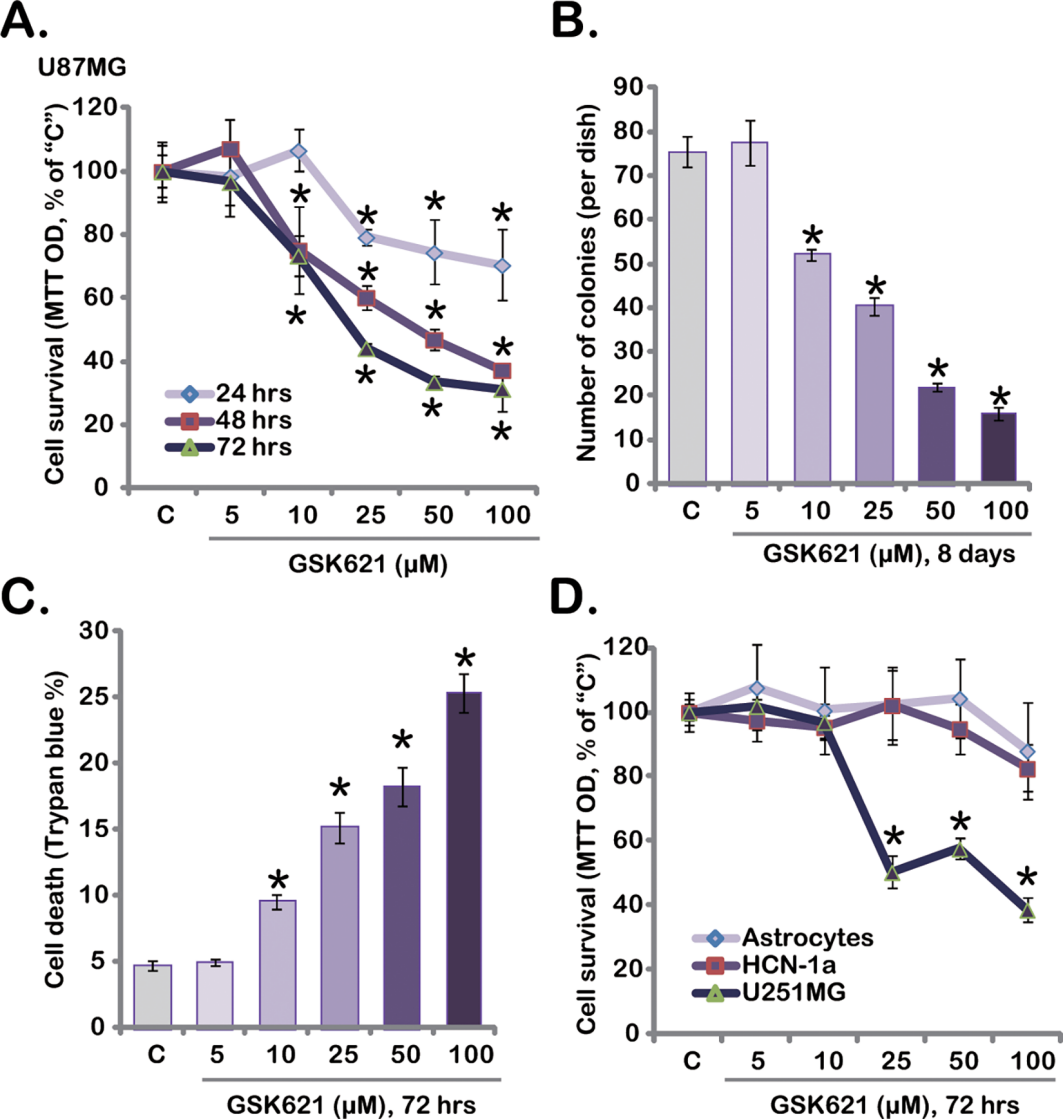
GSK621 inhibits human glioma cell survival. Human glioma U87MG cells (A-C) and U251MG cells (D), as well as HCN-1a neuronal cells (D) or primary human astrocytes (“Astrocytes”, D) were either left untreated (“C”) or treated with applied concentrations of GSK621 for indicated periods of time, cell survival was tested by MTT assay (A and D) and clonogenicity assay (B); Cell death was also tested by the trypan blue staining assay (C). Experiments in this figure were repeated four times, and similar results were obtained. Data were presented as mean ± SD. * p <0.05 vs. “C”.

To access the potential effect of GSK621 on glioma cells, U87MG cells were treated with different concentrations of the AMPK activator. MTT viability assay was performed and the results showed that GSK621 indeed inhibite U87MG cell survival (Fig 1A). The anti-survival activity by GSK621 was both concentration- and time-dependent (Fig 1A). Further, the results of clonogenicity assay showed that GSK621, at 10–100 μM, dramatically decreased the number of viable U87MG colonies (Fig 1B). GSK621’s inhibition on U87MG colony formation was again dose-dependent (Fig 1B). On the other hand, the number of dead (“trypan blue positive”) U87MG cells was increased following GSK621 (10–100 μM) treatment (Fig 1C). We also evaluated the potential activity of GSK621 on other glioma cells. As shown in Fig 1D, treatment of GSK621 decreased the viability OD of U251MG glioma cells. On the other hand, same GSK621 treatment failed to induce significant cytotoxicity to the human HCN-1a neuronal cells (Fig 1D) and primary human astrocytes (Fig 1D). These results suggest that GSK621 inhibits human glioma cell survival.

### 3.2. GSK621 Provokes Apoptosis in Human Glioma Cells

**Fig 2:**
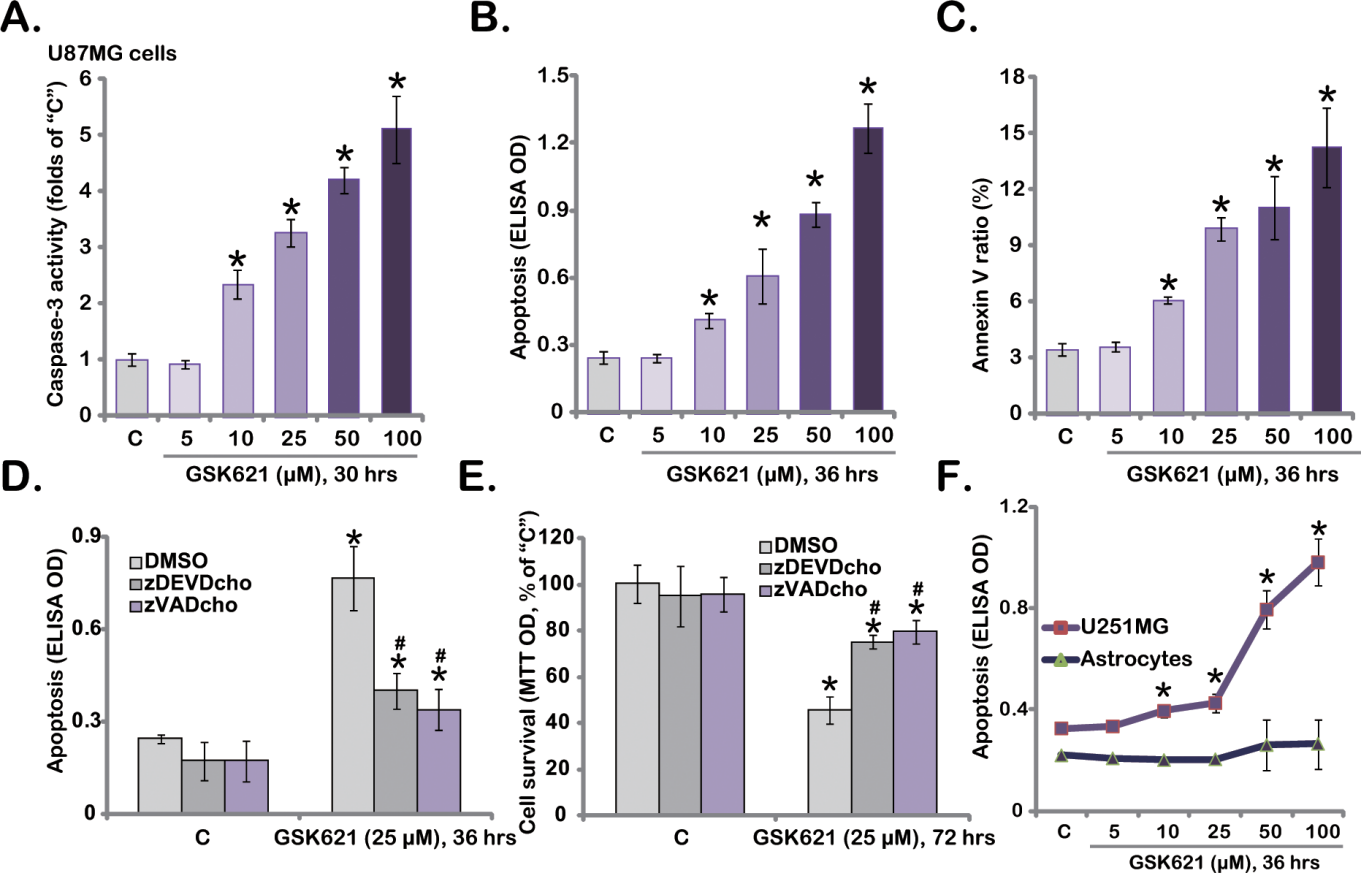
GSK621 provokes apoptosis in human glioma cells. Human glioma U87MG cells (A-E) and U251MG cells (F), as well as primary human astrocytes (“Astrocytes”, F) were either left untreated (“C”) or treated with applied concentrations of GSK621 for indicated periods of time, cell apoptosis was tested by listed assays (A-D, F); For D and E, cells were also pre-treated for 1 hour with the caspase-3 inhibitor z-DEVD-cho (50 μM) or the pan caspase inhibitor zVAD-cho (50 μM), and cell viability (E) was tested as well. Experiments in this figure were repeated three times, and similar results were obtained. Data were presented as mean ± SD. * p <0.05 vs. “C”. ^**#**^ p <0.05 vs. “GSK621” (D and E).

GSK621-induced cytotoxicity in glioma cells could be due to apoptosis induction. Using the methods described previously [4,5,6,7], we tested the potential effect of GSK621 on glioma cell apoptosis. Results showed that, in U87MG cells, GSK621 dose-dependently increased the caspase-3 activity (Fig 2A), histone DNA apoptosis ELISA OD (Fig 2B) and Annexin V percentage (Fig 2C), confirming apoptosis activation. To test the role of apoptosis in GSK621-induced cytotoxicity, caspase inhibitors were applied. Results clearly showed that pretreatment with the caspase-3 inhibitor z-DEVD-cho or the pan caspase inhibitor zVAD-cho attenuated GSK621-induced apoptosis (Fig 2D) and cytotoxicity (Fig 2E, viability reduction) in U87MG cells. These results suggest that GSK621 induces caspase-dependent apoptotic death of glioma cells. Apoptosis ELISA OD results in Fig 2F showed that GSK621 was also pro-apoptotic in U251MG cells, but not in primary human astrocytes.

### 3.3. AMPK Activation Is Required for GSK621-Induced Cytotoxicity in Human Glioma Cells

**Fig 3:**
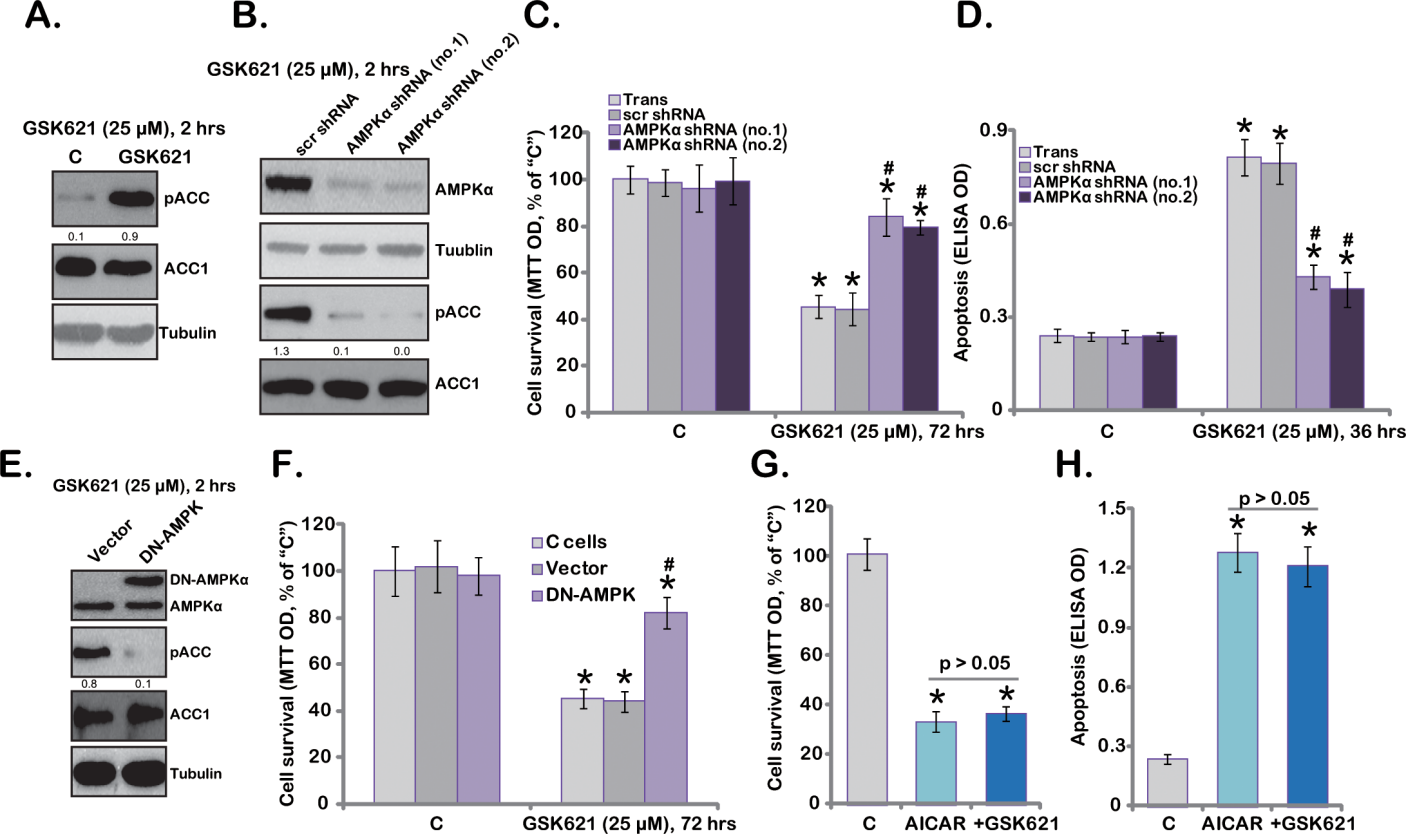
AMPK activation is required for GSK621-induced cytotoxicity in human glioma cells. U87MG cells were either left untreated (“C”) or treated with applied concentrations of GSK621 for 2 hours, phospho- (p-) and regular ACC expressions were tested by Western blots (A). Scramble control shRNA (“scr shRNA”) or AMPKα shRNA (“no.1 or no.2”) expressing stable U87MG cells were treated with GSK621 (25 μM) for applied periods of time, AMPKα and ACC expressions were tested by Western blots (B); Cell survival (C, MTT assay) and apoptosis (D, Histone DNA ELISA assay) were also tested. Stable U87MG cells expressing a dominant negative AMPKα (T172A, “DN-AMPK”) or the empty vector (“Vector”) were treated with GSK621 (25 μM) for applied periods of time, AMPKα and ACC expressions were tested by Western blots (E); Cell survival (F, MTT assay) was also tested. U87MG cells were treated with AICAR (1 mM) or plus GSK621 (25 μM), cell viability (G, MTT assay, 72 hours) and apoptosis (H, Histone DNA ELISA assay, 36 hours) were tested. “Trans” stands for transfection reagents control (C and D). “C cells” stands for “non-transfected control cells” (F). Experiments in this figure were repeated three times, and similar results were obtained. Data were presented as mean ± SD. * p <0.05 vs. “C”. p-ACC (vs. Total ACC) was quantified. ^**#**^ p <0.05 vs. “scr shRNA” (C and D) or “Vector” (F).

GSK621 is a novel AMPK activator [21]. We showed that GSK621 activated AMPK in U87MG cells (Fig 3A). Noted that AMPK activation was detected by the increase of p-acetyl-CoA carboxylase (ACC, Ser-79) (Fig 3A) [7]. To study the connection between AMPK activation and GSK621-mediated cytotoxicity, first we applied the shRNA strategy [7] to knockdown AMPKα in U87MG cells. In the stable U87MG cell lines, the expression of AMPKα was dramatically downregulated (Fig 3B). Two non-overlapping AMPKα shRNAs (no.1/2) were uesd, both of them caused significant AMPKα downregulation (Fig 3B). Consequently, GSK621-induced AMPK activation (p-ACC) was almost blocked in the AMPKα shRNA-expressing cells (Fig 3B). Significantly, AMPKα knockdown also largely inhibited GSK621-induced cytotoxicity (Fig 3C) and apoptosis activation (Fig 3D) in U87 MG cells. These shRNA results suggest that AMPK is required for GSK621-mediated cytotoxicity again U87MG cells.

To further support the requirement of AMPK activation in GSK621’s actions, we next introduced a dominant negative AMPKα (T172A, “DN-AMPK”) [24] into U87MG cells, and stable cell line was established (See methods). Western blot results in Fig 3E confirmed expression of the DN-AMPK in stable U87MG cells, which almost completely blocked AMPK activation (p-ACC) by GSK621. As a result, subsequent cell viability reduction (Fig 3F) and apoptosis activation (Data not shown) by GSK621 were largely attenuated in DN-AMPK-expressing U87MG cells. On the other hand, AICAR, a known AMPK activator [25,26], also induced cytotoxicity (Fig 3G) and apoptosis activation (Fig 3H) in U87MG cells. More importantly, GSK621 failed to induce further cytotoxicity in AICAR-treated U87MG cells. We also repeated the above experiments in U251MG cells, and similar results were obtained (Data not shown). Therefore, these results imply that AMPK activation is required for the GSK621's actions in human glioma cells.

### 3.4. GSK621 Activates AMPK to Inhibit mTOR and Downregulate Tspan8 in Human Glioma Cells

**Fig 4:**
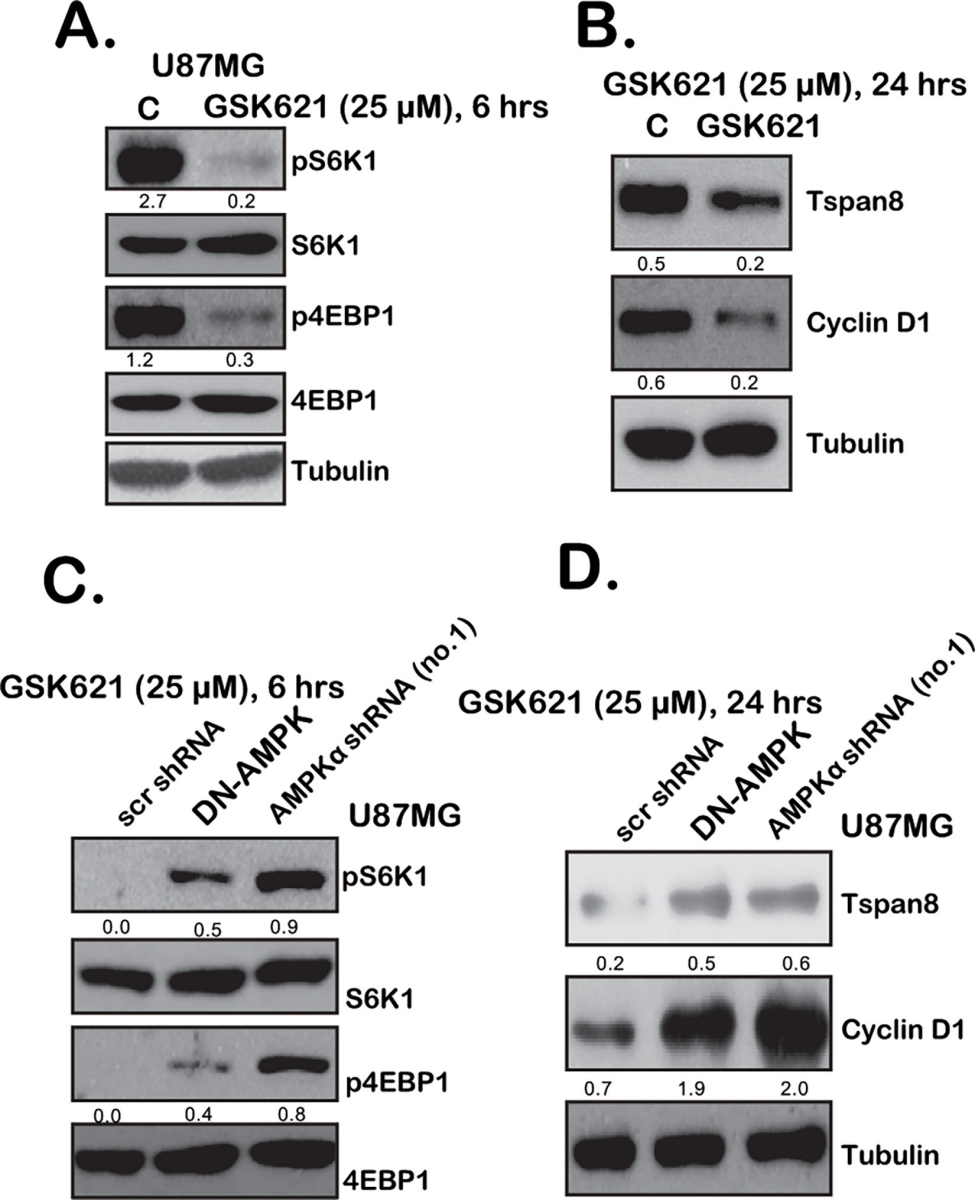
GSK621 activates AMPK to inhibit mTOR and downregulate Tspan8 in human glioma cells. U87MG cells were either left untreated (“C”) or stimulated with GSK621 (25 μM) for applied periods of time, expressions of listed proteins were tested by Western blots (**A** and **B**). Stable U87MG cells with scramble control shRNA (“scr shRNA”), AMPKα shRNA (“-a”) or dominant negative AMPKα (T172A, “DN-AMPK”) were treated with GSK621 (25 μM) for indicated periods of time, expressions of listed proteins were tested by Western blots (**C** and **D**). Indicated protein expression was quantified. Experiments in this figure were repeated three times, and similar results were obtained.

AMPK activation inhibits cancer cells likely via modulating its downstream signaling targets [27,28,29,30]. For example, AMPK could act as a negative regulator kinase of mammalian target of rapamycin (mTOR) [27]. Therefore, we analyzed mTOR activation in GSK321-treated glioma cells. Western blot results in Fig 4A confirmed that p-S6K1 and p-4E-BP1, the indicators of mTOR activation, were largely inhibited following GSK321 treatment in U87MG cells. Further, cyclin D1, the mTOR-regulated gene [31], was also downregulated in GSK321-treated cells (Fig 4B). Interestingly, Tetraspanin 8 (Tspan8), a potential novel oncogene proposed by our previous studies [4,6], was also downregulated by GSK621 (Fig 4B). Similar results were also obtained in U251MG cells (Data not shown). These results demonstrated that GSK621 inhibited mTOR and downregulated Tspan8 in glioma cells. To study the involvement of AMPK in the process, we again utilized the above genetic strategies. Results showed that AMPKα shRNA knockdown or dominant negative mutation rescued mTOR activation (p-S6K1 and p-4E-BP1) in GSK621-treatd U87MG cells (Fig 4C). Further, expressions of cyclin D1 and Tspan8 were also restored in AMPKα-knockdown or mutant cells (Fig 4D). Similar results were also obtained in U251MG cells (Data not shown). These results suggested that AMPK activation is required for GSK621-induced mTOR inhibition and Tspan8 downregulation in human glioma cells.

### 3.5. GSK621 Sensitizes Temozolomide-Induced Anti-Glioma Cell Activity

**Fig 5:**
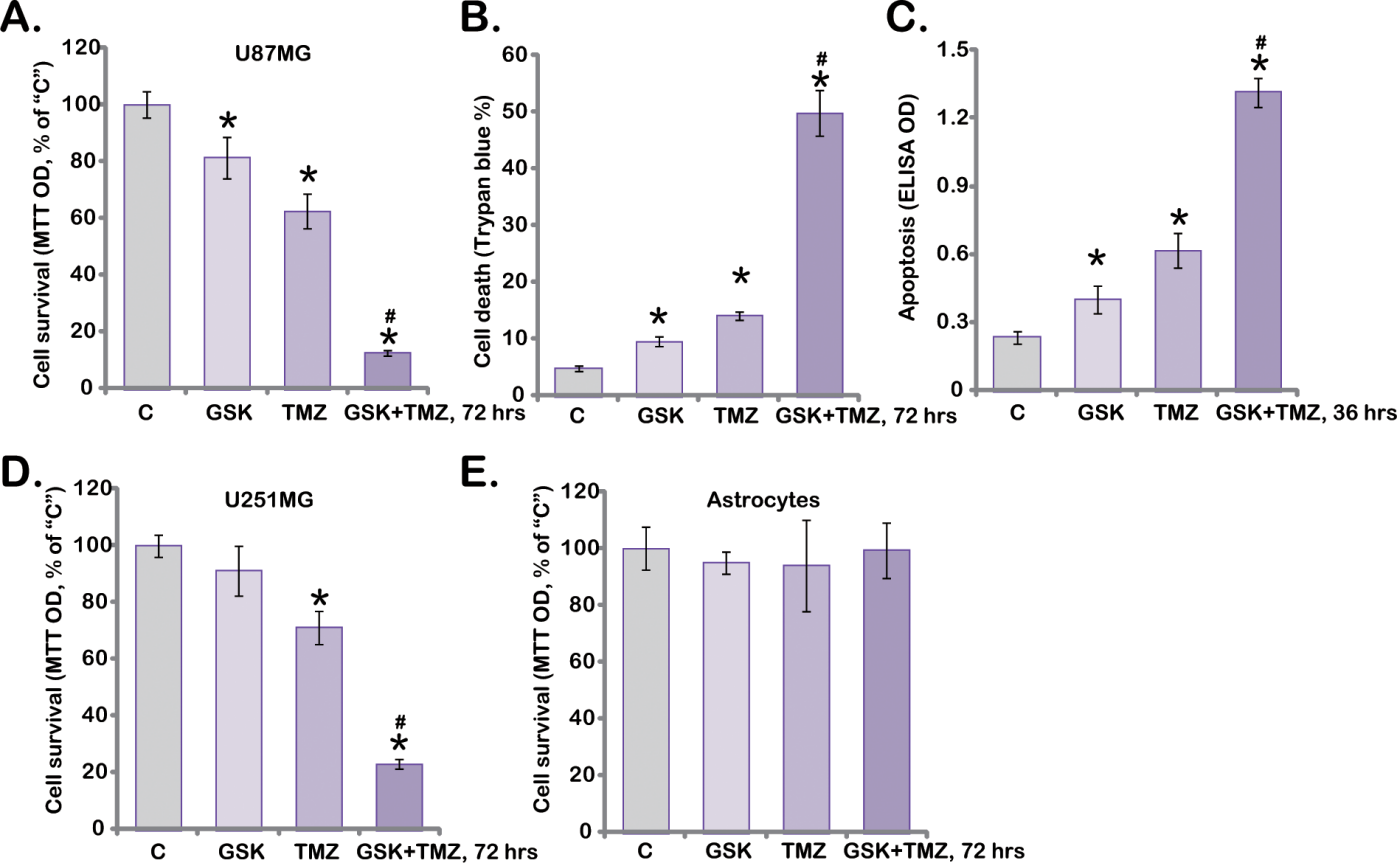
GSK621 sensitizes temozolomide-induced anti-glioma cell activity. U87MG cells (A-C) and U251MG cells (D), as well as primary human astrocytes (E, “Astrocytes”) were treated with temozolomide (“TMZ”, 100 μM) or plus GSK621 (“GSK”, 10 μM) for indicated periods of time, cell survival (A, D and E), cell death (B) and apoptosis (C) were tested by listed assays. Experiments in this figure were repeated three times, and similar results were obtained. Data were presented as mean ± SD. * p <0.05 vs. “C”. ^**#**^ p <0.05 vs. “TMZ” only.

The efficiency of the current standard temozolomide (TMZ) or TMZ-based chemotherapy regimens is moderate, probably due to pre-existing or acquired resistance of human glioma cells [2,32]. The potential effect of GSK621 on TMZ’s activity in glioma cells was then tested. In line with our previous findings [6], treatment with TMZ (100 μM) only induced weak cytotoxicity (Fig 5A and 5B) and apoptosis activation (Fig 5C) in U87MG cells. Significantly, co-treatment with GSK621 dramatically potentiated TMZ’s sensitivity, leading to profound cytotoxicity and apoptosis activation in U87MG cells (Fig 5A–5C). Note that we applied GSK621 at a relatively low concentration (10 μM), which alone only exerted minor actions in U87MG cells (Fig 5A–5C, also see in Fig 1). In U251MG cells, co-treatment with GSK621 (10 μM) again remarkably augmented TMZ’s cytotoxicity (Fig 5D), and the combined activity was dramatically more potent than either single treatment (Fig 5D). Interestingly, same GSK621 plus TMZ co-treatment failed to induce significant cytotoxicity to primary human astrocytes (Fig 5E). Therefore, GSK621 co-treatment dramatically sensitizes TMZ-induced anti-glioma cell activity.

## 4. Discussions

In the present study, we showed that GSK621 activated AMPK signaling and provoked caspase-dependent apoptotic cell death in glioma cells. AMPK inhibition, through AMPKα shRNA knockdown or mutation, dramatically attenuated GSK621’s cytotoxicity against glioma cells. Further, a relatively low concentration of GSK621 significantly potentiated TMZ’s sensitivity and lethality against human glioma cells. Interestingly, no apparent cytotoxicity was noticed in GSK621 (or plus TMZ)-treated normal human astrocytes or neuronal cells. Therefore, GSK621 could be a novel and valuable anti-glioma cell agent.

Existing evidences have shown that mTOR hyper-activation is associated with glioma progression and chemo-resistance [33,34]. Thus, mTOR and it-regulated signalings are important oncotargets for glioma [33,34]. Activation of AMPK is able to inhibit mTOR [27]. AMPK phosphorylates and activates mTOR’s negative regulator protein TSC2 (Tuberous sclerosis protein 2) [27]. Meanwhile, activated AMPK direct phosphorylates and in-activates of Raptor (regulatory associated protein of mTOR), which is a key component of mTOR [35]. In the present study, we showed GSK621 largely inhibited mTOR activation in glioma cells, which could partially explain its anti-glioma cell activity. Significantly, AMPKα mutation or knockdown restored mTOR activation and cyclin D1 expression in glioma cells. These results suggest that GSK621 activates AMPK to inhibit cancer-promoting mTOR signaling in glioma cells.

Tetraspanins, a family of four transmembrane proteins, associate with a large variety of transmembrane and/or cytosolic proteins to promote many pro-cancerous behaviors, including cell migration, adhesion, survival, invasion and proliferation [36,37,38]. Tetraspanin 8 (Tspan8) is uniquely expressed in a small number of normal tissues [39]. Our previous studies have shown that Tspan8 is over-expressed in multiple human malignant glioma tissues, as well as in human glioma cells [4,6], which forms a complex with Rictor (a mTOR component) and integrin α3 to mediate mTOR activation, cell growth, and TMZ resistance [4,6]. In the present study, we showed that GSK621 treatment in glioma cells caused Tspan8 degradation, this could be at least one reason to explain its anti-cancer activity. One key finding of our previous studies is that Tspan8 upregulation is also an important TMZ resistance factor [4]. TMZ’s cytotoxicity could be significantly augmented in Tspan8-silenced glioma cells [4]. In this study, Tspan8 was significantly downregulated following GSK621 treatment in glioma cells, this could explain the dramatic TMZ sensitization effect by the AMPK activator. Future studies will be need to explore the detailed mechanism of Tspan8 downregulation and TMZ sensitization by GSK621.

## 5. Conclusion

In summary, we show that GSK621 inhibits glioma cells via activating AMPK signalings. This novel AMPK activator could be a novel and promising anti-glioma cell agent.
